# Data Mining of Infertility and Factors Influencing Its Development: A Finding From a Prospective Cohort Study of RaNCD in Iran

**DOI:** 10.1002/hsr2.70265

**Published:** 2025-01-24

**Authors:** Hosna Heydarian, Masoumeh Abbasi, Farid Najafi, Mitra Darbandi

**Affiliations:** ^1^ Department of Information Technology Engineering, Industrial and Systems Engineering Faculty Tarbiat Modares University Tehran Iran; ^2^ Department of Health Information Technology, School of Allied Medical Sciences Kermanshah University of Medical Science Kermanshah Iran; ^3^ Research Center for Environmental Determinants of Health (RCEDH), Health Institute Kermanshah University of Medical Sciences Kermanshah Iran

**Keywords:** association rules, clinical decision making, data mining, infertility, PERSIAN cohort

## Abstract

**Background and Aims:**

Infertility, as defined by the World Health Organization, is the inability to conceive after 12 months of regular, unprotected intercourse. This study aimed to identify factors influencing infertility by applying data mining techniques, specifically rule‐mining methods, to analyze diverse patient data and uncover relevant insights. This approach involves a thorough analysis of patients' clinical characteristics, dietary habits, and overall conditions to identify complex patterns and relationships that may contribute to infertility.

**Methods:**

In this study, we examined the impact of lifestyle factors on infertility using machine learning and data mining techniques, specifically Association Rules. The study included a total of 4437 women who participated in the Ravansar Non‐Communicable Disease Cohort study. Among the remaining participants, 434 were infertile. We utilized 38 variables to generate the relevant association rules.

**Results:**

As a result, the analysis reveals that 97% of infertile women are likely to cook for more than 2 h and engage in standing activities. Additionally, 94% of infertile women are likely to have central obesity. Infertile women also have a 73% chance of reusing cooking oil and a 74% chance of consuming fried food at least once a week. The likelihood of infertility increases to 98% among women who use more than 24 eggs per month and to 97% among those who consume moldy jam or syrup. The evaluation of these associations was further supported by measures of support, confidence, and lift.

**Conclusion:**

This study showed that key lifestyle factors linked to infertility, underscoring the role of lifestyle in reproductive health. These findings suggest that targeted interventions and lifestyle changes may help reduce infertility rates. Further research is needed to confirm these associations and investigate the underlying mechanisms.

## 1 Introduction

Fertility has a high value in most cultures, and based on World Health Organization reports, the desire to have a child is one of the most basic human motivations [1]. Infertility is defined as the absence of pregnancy after 12 months or more of intercourse without using any contraceptive methods [2, 3]. In addition, it is described as the inability to have active sexual intercourse after 1 year without using contraceptive methods. Some healthcare providers evaluate women with age more than 35 years old after 6‐month sexual intercourse without contraceptive methods to consider infertility [4]. It is one of the most important problems in reproductive health in developing countries [5]. It can lead to many psycho‐social consequences [6]. Infertility is primary or secondary. Primary infertility is defined as the inability to have a child but secondary infertility is the inability to have a child after a live birth or previous pregnancy [2]. Causes of infertility can be related to males, and females, both of them or unknown (idiopathic infertility). About 40% of infertility problems are related to the male factor, 40% are related to the female factor and about 10% of infertility causes are unexplained [3]. Knowledge about various environmental and lifestyle factors that may affect human health and be related to negative consequences of reproduction is increasing among people and the medical community [7]. Lifestyle factors mean modifiable behavior and ways of living that can affect people's health and sense of well‐being, including fertility [8]. Lifestyle factors negatively or positively affect reproduction health. Normalization of some of these modifiable factors could restore normal egg maturation in women and improve sperm quality in men [9]. These factors may decrease the length of time it takes for a woman to become pregnant and the chances of having a live birth [10].

There is a growing interest among researchers to investigate the role of lifestyle factors in infertility [9, 11, 12]. A study on couples who were trying to conceive showed that after 12 months only 38% of couples who were trying to conceive and had four negative lifestyle factors on fertility had conceived, compared to 52% in couples with three factors with negative effects, 62% in couples with two factors with negative effects and 71% with couples with only one negative lifestyle factor. While in the absence of the negative lifestyle factor, 83% of the couples became pregnant [13].

There is no generally agreed method for developing prediction models from a set of predictive factors [14]. Artificial intelligence (AI) is an effective tool in predicting medical conditions and side effects, and it helps the medical decision‐making process by providing fast, accurate, and interpretable predictions [15, 16].

In the past decade, the rapid advancement of machine learning ‐ a key branch of artificial intelligence ‐ has significantly impacted various research fields, particularly medicine [17]. It has a tremendous impact on data analysis and data science. Machine learning by using new algorithms tries to find hidden patterns in the data and provide a better understanding of the data, and future hypotheses based on past or experimental data and automatically classify data [18].

Regarding the effect of different lifestyle factors on infertility in different research, In this study, we employed association rules to uncover hidden relationships between lifestyle factors and infertility in women from the Ravansar cohort. Our goal was to identify connections that could either confirm or challenge previous research findings or reveal new insights that may have been overlooked by experts.

## 2 Methods

The data mining process involves several key steps. Initially, data cleaning is performed. Following this, data integration occurs as data is collected from various sources. Data reduction is then applied to ensure data quality, selecting only the relevant information. This is followed by data transformation, where the data is summarized or aggregated. Data mining is then conducted to uncover patterns. Subsequently, pattern evaluation is carried out to assess the findings. Finally, the knowledge is represented and presented to provide insights from the data [19, 20]. So, This study was conducted in multiple stages as follows to identify lifestyle factors related to infertility:

### 2.1 Ethical Consideration

All methods were carried out according to relevant guidelines and regulations. All the participants were provided oral and written informed consent. The Ethics Committee of Kermanshah University of Medical Sciences approved the study (KUMS. REC.1394.318).

### 2.2 Data Set

This data was collected at one point in time for 10,047 patients in the west of Iran. It was received from the Kermanshah School of Health. Ravansar Non‐Communicable Disease (RaNCD) cohort in people aged between 35 and 65 years old in Ravansar. In this study, the inclusion criteria were married women, and the exclusion criteria were pregnant and postmenopausal women. Both oral and written consent was obtained from all participants in the cohort study. A full description of the RaNCD study has been published [21].

The demographic information for patients in the RaNCD cohort study was collected by experts using four different questionnaires. Additionally, measurements of blood pressure, height, weight, and BMI were taken according to the cohort profile. Socioeconomic status (SES) was determined using 18 items and analyzed with principal component analysis (PCA), and then categorized into three ordered groups from lowest to highest. Smoking was defined as smoking at least 100 cigarettes during their lifetime, while alcohol consumption was defined as drinking 200 mL of beer or 45 mL of liquor at least once a week for a minimum of 6 months.

To examine physical activity, the cohort questionnaire included 22 questions that categorized activity levels into three groups based on MET/hour per day: low (24–36.5), medium (36.6–44.4), and high (≥ 44.5). Nutritional information was gathered using a Food Frequency Questionnaire (FFQ) with 118 items to assess dietary intake and energy levels. Biochemical markers were measured after a 12‐h fast and included creatinine (Cr), blood urea nitrogen (BUN), high‐density lipoprotein cholesterol (HDL‐C), low‐density lipoprotein cholesterol (LDL‐C), triglycerides (TG), total cholesterol (T‐C), fasting blood sugar (FBS), γ‐glutamyltransferase (GGT), alanine aminotransferase (ALT), and aspartate aminotransferase (AST).

Obesity phenotypes were assessed using BMI (kg/m²), with classifications as normal weight (BMI: 18.5–24.9), overweight (BMI: 25–29.9), and obese (BMI: ≥ 30). Metabolic health was defined according to the International Diabetes Federation (IDF) criteria for metabolic syndrome (MetS). Four obesity‐metabolic syndrome phenotypes were categorized based on BMI and MetS status: metabolically healthy nonobese (MHNO), metabolically healthy obese (MHO), metabolically unhealthy nonobese (MUNO), and metabolically unhealthy obese (MUO). In this research, “infertility” refers to primary infertility, and “infertile women” denotes women whose infertility has been diagnosed by a physician. Descriptions of these variables are provided in Table 1.

**Table 1:** Features and their description.

	Features	Description and Frequency	
1)	Sleep Duration 24 h	Useful sleep (7–8 h), (*n* = 153)	
		Sleep deprivation(less than 7 h), (*n* = 181)	
		Oversleeping (more than 8 h), (*n* = 100)	
2)	House cleaning	Less than or equal to 3 h, (*n* = 62)	
		More than 3 h, (*n* = 372)	
3)	Physical activity	Less than or equal to 6 h, (*n* = 136)	
		More than 6 and less than 12 h, (*n* = 258)	
		Greater than or equal to 12 h, (*n* = 40)	
4)	Age (year)	1. 35–44, (*n* = 2535) 2. 45–54, (*n* = 1691) 3. More than 55, (*n* = 1357)	
5)	Added sugar	Less than or equal to 100 calories, (*n* = 251)	
		Between 100 and 220 calories, (*n* = 2743)	
		More than 220 calories, (*n* = 2289)	
6)	Saturated FAT	Allless than 21 g, (*n* = 5283)	
7)	Socioeconomic status	First quantile, (*n* = 96)	
		Second quantile, (*n* = 62)	
		Third quantile, (*n* = 81)	
		Fourth quantile, (*n* = 101)	
		Fifth quantile, (*n* = 94)	
8)	Salt use	Low‐salt consumption, (*n* = 659)	
		Medium‐salt consumption, (*n* = 1195)	
		High salt consumption, (*n* = 3429)	
9)	BMI (kg/m2)	BMI: < 18.5, (*n* = 175)	
		BMI: 18.5 – 24.9, (*n* = 2096)	
		BMI: 25 – 34.9, (*n* = 1558)	
		BMI: 35 – 39.9, (*n* = 1080)	
		BMI >= 40, (*n* = 374)	
10)	Wrist circumference (cm)	Normal, (*n* = 4692)	
		Abnormal, (*n* = 591)	
11)	Now pregnant	No pregnant (*n* = 5186)	
		Pregnant (*n* = 97)	
12)	Used oil type	Frying‐liquid‐oil, (*n* = 1426)	
		Liquid, (*n* = 1738)	
		No‐frying, (*n* = 369)	
		other, (*n* = 407)	
		Semi‐solid, (*n* = 1025)	
		Solid‐oil, (*n* = 318)	
13)	Re use oil	1. No	7194
		2. Yes	2853
14)	Re use mold	1. No	4880
		2. Yes	5167
15)	LDL cholesterol	Normal (*n* = 1469)	
		High (*n* = 2343)	
		Very high (*n* = 1471)	
16)	Grilled food	Never, (*n* = 106)	
		daily, (*n* = 920)	
		1_3_per_week, (*n* = 3112)	
		1–3‐per‐month, (*n* = 1115)	
		Less‐than‐once‐a‐month, (*n* = 30)	
17)	Fried food	Never, (*n* = 106)	
		Daily, (*n* = 1946)	
		1_3_per_week, (*n* = 2164)	
		1–3‐per‐month, (*n* = 1001)	
		Less‐than‐once‐a‐month, (*n* = 66)	
18)	Pregnancy hypertension	No 5037	
		Yes 246	
19)	Pregnancy diabetes	No 5068	
		Yes 215	
20)	Menstruation	No 44	
		Yes 5239	
21)	Death childbirth	No 4853	
		Yes 430	
22)	Tubectomy	No 3570	
		Yes 1713	
23)	Hysterectomy	No 5069	
		Yes 214	
24)	Infertility	No 4849	
		Yes 434	
25)	Cervical or breast Ca screening	No 2514	
		Yes 2769	
26)	Breast exam	No 4637	
		Yes 646	
27)	Mammography	No 4654	
		Yes 629	
28)	Pap smear	No 2633	
		Yes 2650	
29)	Menstruation start age	1. <= 11, *n* = 297	
		2. 12–15, *n* = 4313	
		3. >= 16, *n* = 63	
30)	Number of pregnancy	0. 465 No	
		1. 1800 less is equal to three times	
		2. 2512 between and equal to 4 and 8 times	
		3. 506 between and equal to 9 and 19 times	
31)	Number of alive child birth	0. 53 The child never survived	
		1. 2584 survived less than three times	
		2. 2282 survived 4–8 times	
		3. 364 survived between 9 and 17 times	
32)	First alive child birth age	1. <= 14, *n* = 101	
		2. 15–27, *n* = 4700	
		3. 28–46, *n* = 482	
33)	First pregnancy age	1. <=13, *n* = 100	
		2. 14–26, *n* = 4700	
		3. 27–45, *n* = 483	
34)	Use infertility drug	No 5001	
		Yes 279	
35)	Use contraceptive drug	No 1223	
		Yes 4060	
36)	Menopause age	1. 37–44, *n* =﻿﻿ 30 2. 45–55, *n* = 394 3. >= 55, *n* = 924	
37)	Vegetable	First group: <= to 90 g ‐ 102 people Second group: Between 90 and 150 g ‐ 308 people The third group: More than 150 g ‐ 9637 people	

### 2.3 Data Preprocessing

The raw data was messy and unorganized, so it was cleaned up and then converted into a more organized and manageable format by categorizing continuous values into discrete intervals. Because outlier values were occasionally present in the data set, they were first removed, and the variable was handled similarly to how variables with missing values were treated. For example, the age data for women was expected to fall between 35 and 65; thus, an age of 90 indicated an outlier.

To address missing values, an appropriate method—mean, mode, or deletion—was applied to each variable. The mean is suitable for normally distributed (symmetric) data, while the median is preferable for asymmetric distributions. For instance, the median was used to fill in missing values for the “Use oil type” variable due to its asymmetric distribution. Conversely, for the “Wrist circumference” variable, which had a symmetric distribution, the mean was applied to impute missing values and categorized the data as either Normal or Abnormal [22].

The next step was to discretize the variables. All variables were categorized based on references or data analysis. For example, the WHO reference and global reports were used to discretize the BMI variable, while data analysis was applied to categorize the “age of first pregnancy” and the “number of abortions” variables.

### 2.4 Data Processing

Data mining is the computational process of discovering patterns and extracting valuable insights from large datasets. It involves analyzing data from multiple perspectives and summarizing it to reveal previously unknown relationships and trends. These insights can then be used for informed decision‐making and future trend predictions. Key goals of data mining include discovering hidden patterns, forecasting future trends based on historical data, classifying data into predefined categories, clustering similar data points to reveal underlying structures, and detecting anomalies to identify outliers or unusual data points that deviate from the norm [23].

Association rules are a technique used to discover hidden relationships within datasets. These rules identify sets of frequently occurring items that reveal underlying relationships in the data. In this paper, association rules are employed to analyze and predict patient behavior and lifestyle. Agrawal, Imielinski, and Swami first introduced association rules in 1993. These rules are derived from data through repetitive If–then patterns and are evaluated using key metrics called support and confidence. These metrics help to identify and understand relationships within the data [24].

Support shows the probability of occurrence of an item and its evidentness in the entire target data, while confidence introduces the probability of finding If–then statements next to each other. The formulas are provided below:

$$\mathrm{Sup}(A)=\frac{\mathrm{count}(A)}{\mathrm{total number of transactions}}$$

$$\mathrm{Conf}(A\to B)=\frac{\text{sup}(A\cup B)}{\text{sup}(A)}$$

According to all these interpretations, there is a third criterion called lift, which is the best indicator for the correctness of the results and their evaluation. Actual and expected confidence can be compared. Lift shows us the number of times the If–then statement was correctly recognized.

$$\mathrm{Lift}(A\to B)=\frac{\mathrm{Conf}(A\to B)}{\mathrm{Sup}(A)\mathrm{Sup}(B)}$$

To produce association rules, the Apriori algorithm was used. In 1993, this algorithm was proposed to generate similar items and related rules [25]. The advantage of choosing this algorithm was due to its compatibility with the large volume of data. It also has high accuracy and reliability in producing results [26]. However, the Apriori algorithm loads the candidate set with as many subsets as possible before each scan of the database, causing over‐scanning and reducing overall performance. The algorithm assumes that the database is permanently in memory. Moreover, both the time and space complexity of this algorithm are exceptionally high. for implementation of this algorithm, minimum support and minimum confidence must be established and defined by experts and a review of relevant literature.

To streamline rule generation, items should be filtered to exclude those with support and confidence below a specified threshold, leading to an exponential reduction in production rules. The process begins by examining the occurrence probability of individual items, and progressively analyzing item sets with higher occurrence probabilities. This approach, known as the Apriori algorithm, leverages information about frequent item collections. It employs a bottom‐up strategy where items are expanded collectively, a process referred to as item production. The algorithm utilizes a breadth‐first search and a hash tree structure to efficiently count candidate items [27].

The algorithm ends when there are no items that can be checked. In the end, the database checks all generated rules and transactions to find the set of repeated items among the candidates

The algorithm generates candidate itemsets of length k from the item set of length k‐1, then prunes candidates with rare subpatterns. According to the downward closure lemma, the candidate set inherently contains all k frequent item sets. Subsequently, it scans the transaction database to determine the set of frequent occurrences among the candidates unequivocally.

**Table:** 

Apriori(T, ε) L_1_ ← {large 1 ‐ itemsets} k ← 2 while L_k−1_ is not empty C_k_ ← Apriori_gen(L_k−1_, k) for transactions t in T D_t_ ← {c in C_k_: c ⊆ t} for candidates c in D_t_ count[c] ← count[c] + 1 L_k_ ← {c in C_k_: count[c] ≥ ε} k ← k + 1 return Union(L_k_) Apriori_gen(L, k) result ← list() for all p ⊆ L, q ⊆ L where p_1_ = q_1_, p_2_ = q_2_,…, p_k‐2_ = q_k‐2_ and p_k‐1_ < q_k‐1_ c = p ∪ {q_k‐1_} if u ⊆ c for all u in L result. add(c) return result.

## 3 Results

For the analysis, among all the RaNCD patients reviewed, 5283 were women, including 4,436 who were married. Of these women, 97 were pregnant and 6 were in menopause; these cases were excluded from the study. Additionally, 434 (14.5%)women were identified as infertile, of whom 284 (65%) were receiving fertility medications. The general characteristics of the participants are detailed in Table 2, and the participant selection process is illustrated in Flowchart 1.

**Table 2:** Basic characteristics of participants.

Variables	Mean
Age of women	43.5
Weight of Infertiles (kg)	69.88
Physical activity of women	11.78
Pregnancy number	4.45
Abortion number	0.31
First pregnancy age (year)	18.4

**Figure 1:**
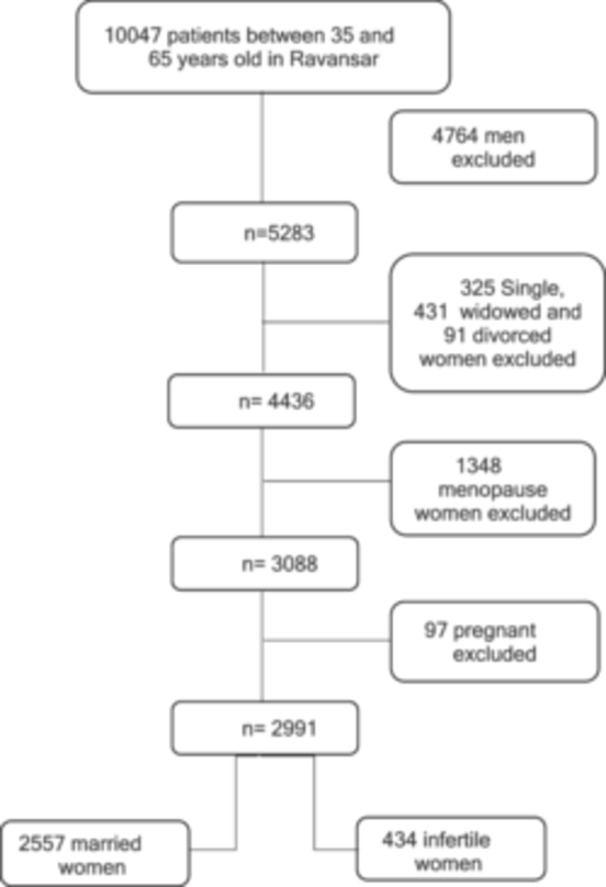
Flowchart of Female participants.

**Figure 2:**
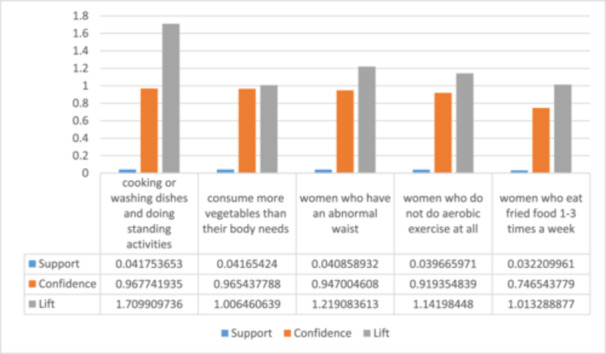
Chart of rules that infertility is in part Antecedent.

Based on expert's views, The minimum support was 0.001 and the minimum confidence was 0.6. The minimum lift is 1 and rules with lift > = 1 were acceptable to us. This approach ensured that our parameter choices were grounded in established practices, enhancing the validity of our findings (Figure 2).

**Figure 3:**
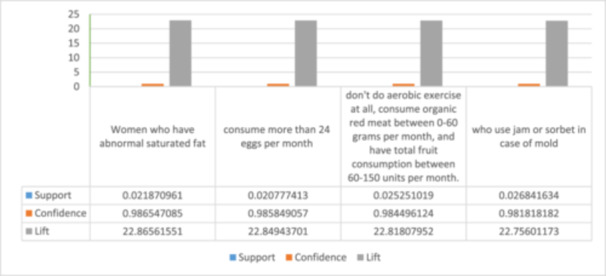
Chart of rules that infertility is in part consequent.

APRIORI algorithm was used to extract association rules with a minimum support value of 0.001 and minimum confidence of 0.6. Only 2% of infertility cases had amenorrhea, which was related to infertile women who had gone through menopause. 60% of them had low HDL cholesterol. Gynecologists checked extracted rules and the approved rules are mentioned in Table 3 and Table 4 in the two categories of significant rules (Figure 3).

**Table 3:** The rules that infertility is in part Antecedent.

No	Rule	Support	Confidence	Lift	Count	Adjust
						Support for rule
1	Infertile women who spend more than 2 h for cooking or washing dishes and doing standing activities.	0.04175	0.96774	1.70990	420	**0.967742**
2	Infertile women who consume more vegetables than their body needs in a month. More than 150 g (in terms of calories, 25 calories are equivalent to 1 unit of vegetables).	0.04165	0.96543	1.00646	419	**0.965438**
3	Infertile women who have an abnormal waist (central obesity).	0.04085	0.94700	1.21908	411	**0.947005**
4	Infertile women who have a total of 12 h of physical activity and more than 2 h of brisk walking.	0.04056	0.94009	1.07777	408	**0.940092**
5	Infertile women who do not do aerobic exercise at all.	0.03966	0.91935	1.14198	399	**0.919355**
6	Infertile women who consume organic red meat between 0 and 60 g per month and it are in the low consumption range.	0.03897	0.90322	1.02165	392	**0.903226**
7	Infertile women who eat fried food 1–3 times a week.	0.03220	0.74654	1.01328	324	**0.746544**
8	Infertile women who used oil at least once.	0.03171	0.73502	1.02760	319	**0.735023**

**Table 4:** The rules that infertility is in part consequent.

No	Rule	Support	Confidence	Lift	Count	Adjust
						Support for rule
1	Women who have abnormal saturated fat, have their first live child at the age of 28–46, and also have an abnormal weight.	0.02187	0.98654	22.86561	220	**0.506912**
2	Those who consume more than 24 eggs per month and eat fried food 1–3 times per week.	0.02077	0.98584	22.84943	209	**0.481567**
3	Those who don't do aerobic exercise at all, consume organic red meat between 0 and 60 grams per month, and have total fruit consumption between 60 and 150 units per month.	0.02525	0.98449	22.81807	254	**0.585253**
4	Those who spend more than 2 h for cooking and washing dishes, and who spend more than 2 h on their feet, and who use jam or sorbet in case of mold, and who consume less than 600 grams of dairy products.	0.02684	0.98181	22.75601	270	**0.62212**

## 4 Discussion

In this study, we examined the influencing factors in infertility and the conditions of infertility using association rules. Using Machine‐learning methods to develop improved prediction models for clinical decision‐making is increasing [28]. Based on the results, physical activity, obesity, and nutritional factors were associated with infertility with different possibilities.

More than 90% of infertile women had intense physical activity. There is evidence that physical activity and infertility are related. Exercise may play a role in decreasing the body's energy balance, and it can lead to amenorrhea and irregular ovulation [12]. Doing balanced exercise regularly is recommended, and may improve fertility in infertile couples. Because intense physical activity may expose a person to infertility [29].

In some studies, age has been known as an effective factor in infertility, so the incidence of fetal abnormalities and recurrent miscarriage increases with the increasing age of the mother [30]. The fertility peak in men is before 35 years old and in women is before 30 years old [29], and after turning 40, the chance of fertility decreases [31]. The results of various research show that the chance of pregnancy with assisted reproductive treatments (ART), and live birth decreases with age [32, 33]. In the present study, age was not extracted as an important factor in association rules. It seems that the age range of participants and their age classification method can be the main cause of this. Because the participants in the cohort study were between 35 and 65 years old, and the age categories were 35–44, 45–54, and 55–65 years. As mentioned above, even in the first category, we are facing a decreased fertility rate.

Weight can have a significant impact on health, including heart disease, diabetes, and infertility [34]. In addition, research shows that maintaining a healthy weight helps to prevent hormonal imbalance, and even weight loss of obese infertile women contributes to better treatment results [29]. In this regard, the results of our study showed that abnormal saturated fats, abnormal weight (obesity), and especially central obesity are effective factors in infertility so 94% of those with infertility had an abnormal waist circumference (Central obesity).

Another factor that is related to the increase in general and central obesity is the consumption of fried foods [35, 36]. The relationship between the consumption of fried food and the occurrence of heart disease and gestational diabetes has been investigated [37, 38]. The study conducted by Çekici in 2019 showed that the consumption of trans fatty acids by women increases the risk of infertility due to ovarian factors [39]. A cohort study conducted by Chavarro in 2007 showed that consuming trans‐unsaturated fats instead of carbohydrates or unsaturated fats that are commonly found in non‐hydrogenated vegetable oils may increase the risk of infertility due to ovarian problems [40]. The reviewed studies were about the relationship between trans‐fat and infertility. Our question in the cohort questionnaire was about frying oils. Knowing the amount of trans‐fat consumed in frying oil and its other compounds can help us better conclude its impact on infertility. It seems investigating the impact of various frying oils on infertility can be a research.

**Figure 4:**
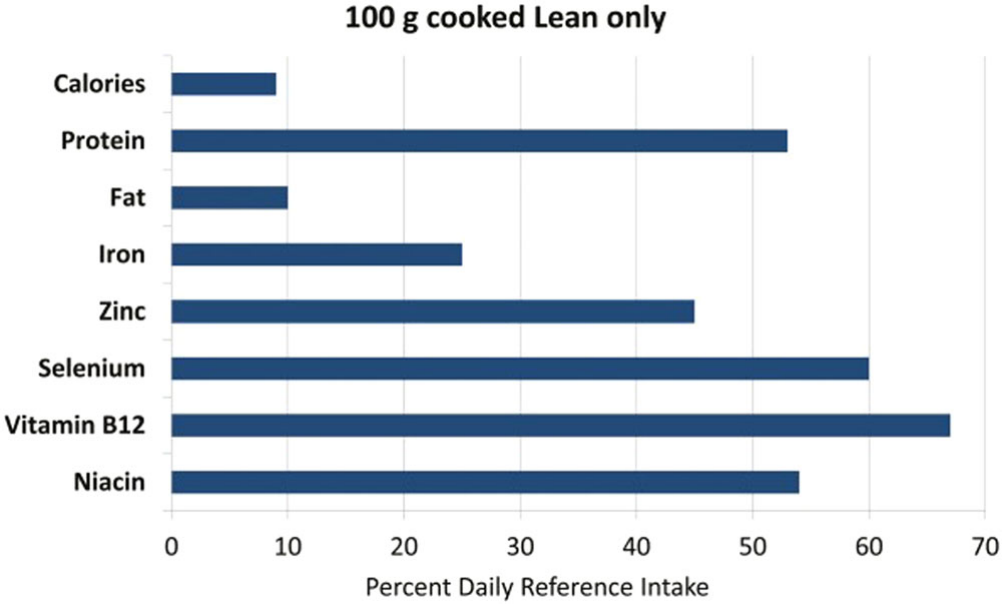
Some of the micronutrients [45].

Recently, protein consumption and its effect on women's reproductive health have received special attention due to its possible association with environmental pollution [41, 42]. NHS ΙΙ suggests that the consumption of animal proteins in healthy women exposes them to ovarian problems compared to women who consume vegetable proteins [43]. However, a lack of animal proteins can cause a deficiency of important micronutrients such as vitamin B12, zinc, calcium, and selenium [44]. Several of these micronutrients are among the most commonly deficient micronutrients in the world, which can be increased by consuming just a few ounces of beef per week (Figure 4) [45].

There is no strong evidence linking the consumption of red or processed meat to cancer, but the available evidence does not mean that eating any amount of red meat is healthy. Like any other food, high consumption of this food is probably associated with adverse health effects [46]. The results of this study showed that 90% of those who have infertility consume organic red meat between 0 and 60 g per month, which is in line with the mentioned studies.

## 5 Limitation

This study has several strengths and limitations. We used a big data set with high‐quality data. Experts perform regular quality control of data. The cohort data is related to one center (Ravanser). Using data from more centers will produce more reliable results. One of our limitations was examining Simpson's paradox for each category of variables. Fortunately, we did not reach different results. Simpson's paradox refers to a phenomenon in statistics and probability in which when data are examined in categories, they show different results than when they are examined as a whole. Usually, one of the reasons for this difference is the imbalance of the categories.

## 6 Conclusion

This study utilized data mining techniques to identify lifestyle factors associated with infertility among women aged 35–65 in Kermanshah, Iran. Employing the Apriori algorithm, we uncovered significant association rules linking intense physical activity, poor dietary habits (notably high fried food consumption), and obesity metrics to infertility. These findings not only deepen our understanding of the lifestyle influences on infertility but also underscore the importance of targeted public health interventions. By addressing these modifiable risk factors, we can potentially improve reproductive health outcomes for women. Future research should expand on these findings and explore longitudinal effects and causative relationships to further enrich the field of infertility studies.

## Author Contributions

**Masoumeh Abbasi:** writing–original draft, writing–review and editing, validation, visualization. **Hosna Heydarian:** writing–original draft, formal analysis, data curation, supervision, project administration, visualization, methodology, writing–review and editing, conceptualization, investigation, software. **Farid Najafi:** validation, project administration, resources, supervision, investigation, visualization. **Mitra Darbandi:** validation, visualization.

## Ethics Statement

Ethical approval for the study was obtained from the Ethical Committee of Kermanshah University of Medical Sciences. All methods were carried out in accordance with relevant guidelines and regulations. Informed consent was obtained from all participants. The Research and Technology Deputy and the Ethical Committee of Kermanshah University of Medical Sciences approved the study protocol (Ethical Number: KUMS. REC.1394.318). Participants provided oral and written informed consent.

## Conflicts of Interest

The authors declare that they have no competing interests.

## Data Availability

The authors confirm that the data supporting the findings of this study are available from the corresponding author upon reasonable request.
